# A One Health Study of the Genetic Relatedness of *Klebsiella pneumoniae* and Their Mobile Elements in the East of England

**DOI:** 10.1093/cid/ciz174

**Published:** 2019-03-07

**Authors:** Catherine Ludden, Danesh Moradigaravand, Dorota Jamrozy, Theodore Gouliouris, Beth Blane, Plamena Naydenova, Juan Hernandez-Garcia, Paul Wood, Nazreen Hadjirin, Milorad Radakovic, Charles Crawley, Nicholas M Brown, Mark Holmes, Julian Parkhill, Sharon J Peacock

**Affiliations:** 1 Department of Pathogen Molecular Biology, London School of Hygiene & Tropical Medicine, Hinxton; 2 Wellcome Trust Sanger Institute, Wellcome Trust Genome Campus, Hinxton; 3 Center for Computational Biology, Institute of Cancer and Genomic Sciences, University of Birmingham, Cambridge; 4 Department of Medicine, University of Cambridge, Addenbrooke’s Hospital, Cambridge; 5 Cambridge University Hospitals National Health Service Foundation Trust, Cambridge; 6 Clinical Microbiology and Public Health Laboratory, Public Health England, Cambridge; 7 Department of Veterinary Medicine, University of Cambridge, United Kingdom; 8 Royal (Dick) School of Veterinary Studies, University of Edinburgh, United Kingdom

**Keywords:** *Klebsiella pneumonia*, One Health, genomics, antimicrobial resistance, surveillance

## Abstract

**Background:**

*Klebsiella pneumoniae* is a human, animal, and environmental commensal and a leading cause of nosocomial infections, which are often caused by multiresistant strains. We evaluate putative sources of *K. pneumoniae* that are carried by and infect hospital patients.

**Methods:**

We conducted a 6-month survey on 2 hematology wards at Addenbrooke’s Hospital, Cambridge, United Kingdom, in 2015 to isolate *K. pneumoniae* from stool, blood, and the environment. We conducted cross-sectional surveys of *K. pneumoniae* from 29 livestock farms, 97 meat products, the hospital sewer, and 20 municipal wastewater treatment plants in the East of England between 2014 and 2015. Isolates were sequenced and their genomes compared.

**Results:**

*Klebsiella pneumoniae* was isolated from stool of 17/149 (11%) patients and 18/922 swabs of their environment, together with 1 bloodstream infection during the study and 4 others over a 24-month period. Each patient carried 1 or more lineages that was unique to them, but 2 broad environmental contamination events and patient–environment transmission were identified. *Klebsiella pneumoniae* was isolated from cattle, poultry, hospital sewage, and 12/20 wastewater treatment plants. There was low genetic relatedness between isolates from patients/their hospital environment vs isolates from elsewhere. Identical genes encoding cephalosporin resistance were carried by isolates from humans/environment and elsewhere but were carried on different plasmids.

**Conclusion:**

We identified no patient-to-patient transmission and no evidence for livestock as a source of *K. pneumoniae* infecting humans. However, our findings reaffirm the importance of the hospital environment as a source of *K. pneumoniae* associated with serious human infection.

## Teaser

A One Health genomic survey of *Klebsiella pneumoniae* demonstrated transmission between patients and their ward environment but no evidence to implicate livestock as a source of *K. pneumoniae* or their mobile genetic elements encoding antibiotic resistance.

*Klebsiella pneumoniae* is a major cause of nosocomial infections worldwide, the public health importance of which has been amplified by the increasing prevalence of multidrug-resistant *K. pneumoniae* carriage and infection [1–3]. Resistance can be attributed to the global dissemination of extended-spectrum β-lactamase (ESBL)-producing *K. pneumoniae* and the subsequent emergence of isolates with resistance to the carbapenem drugs and colistin. Control of human *K. pneumoniae* infection requires an understanding of the potential sources from which infecting organisms are acquired. This is complex since *K. pneumoniae* can persist in a broad range of reservoirs including the hospital environment [4–7], retail meat [8, 9], livestock [10, 11], and wastewater [12, 13].

Attributing sources of *K. pneumoniae* that cause infection requires the sampling of multiple reservoirs at the same time and in the same place and comparing these using whole genome analyses. Previous genomic studies have largely focused on transmission in high-risk settings such as intensive care units. These studies have identified *K. pneumoniae* carriage as a significant risk factor for infection [14, 15]. Accurate interpretation of transmission also requires an understanding of whether specific reservoirs and individual samples contain more than 1 *K. pneumoniae* lineage. A previous study identified limited within-host diversity in 20% of patients based on sequencing of up to 3 *K. pneumoniae* colonies from 40 patients [15].

Here, we explore the genetic relatedness of *K. pneumoniae* isolated from the same and different reservoirs within a defined geographic region through an investigation of isolates from a patient cohort, their hospital environment, livestock, municipal wastewater, and hospital sewage in the same geographic region of England.

## METHODS

### Patient Recruitment and Sampling

A prospective longitudinal study was conducted in 2 adult hematology wards at the Cambridge University Hospital National Health Service Foundation Trust (CUH) between May 2015 and November 2015. Patients were enrolled after informed written consent was obtained, after which stool samples were requested on the day of admission, every week thereafter, and at discharge and cultured for *K. pneumoniae*. Environmental sampling for *K. pneumoniae* was performed throughout the study, details of which are provided in the Supplementary Material. All blood cultures from patients on the 2 study wards between May 2014 and May 2016 were identified and stored isolates were obtained. Wastewater was sampled from the main sewer of CUH on 4 spaced occasions between September 2014 and December 2015. A cross-sectional survey was conducted between June 2014 and January 2015 to isolate *K. pneumoniae* from raw and treated wastewater at 20 municipal wastewater treatment plants in the East of England. Ten plants were located downstream of acute hospitals and 10 did not directly receive hospital waste. A cross-sectional survey was conducted between August 2014 and April 2015 to isolate *K. pneumoniae* from livestock at 29 farms in the East of England (10 cattle [5 beef/5 dairy], 10 pig, and 9 poultry [4 chicken/5 turkey]. The Supplementary Material provides further details of the study design, sample collection, and laboratory methods for bacterial culture, identification, and antimicrobial susceptibility testing.

### Whole-genome Sequence Analysis

Bacterial DNA was extracted using the QIAxtractor (QIAgen) and sequenced on an Illumina HiSeq2000 (Illumina, San Diego, CA). The *de novo* assembly of short-read data was performed as previously described [16, 17]; assemblies were annotated using Prokka [18], and a core genome alignment was produced using Roary. Further analysis was performed for sequence types (STs) ST307, ST268, ST6, ST34, ST3010, and ST2585. Isolates belonging to these 6 STs were each mapped against a study isolate with the best N50 for that ST. Mapping was performed using SMALT [19] and recombination was removed using Gubbins [20]. Single-nucleotide polymorphisms (SNPs) were extracted using an in-house tool [21]. Maximum likelihood trees were created using RAxML with 100 bootstraps and a midpoint root. Phylogenetic trees and associated metadata were visualized using iTOL [22] and Figtree [23]. Multilocus STs, antimicrobial resistance determinants, virulence factors, and plasmids were identified. A detailed description of the rationale for selecting isolates for sequencing and genomic analyses is provided in the Supplementary Material.

## RESULTS

### Isolation of *K. pneumoniae* From Stool, Blood, and the Environment of a Hospital Cohort

**Figure 1.**
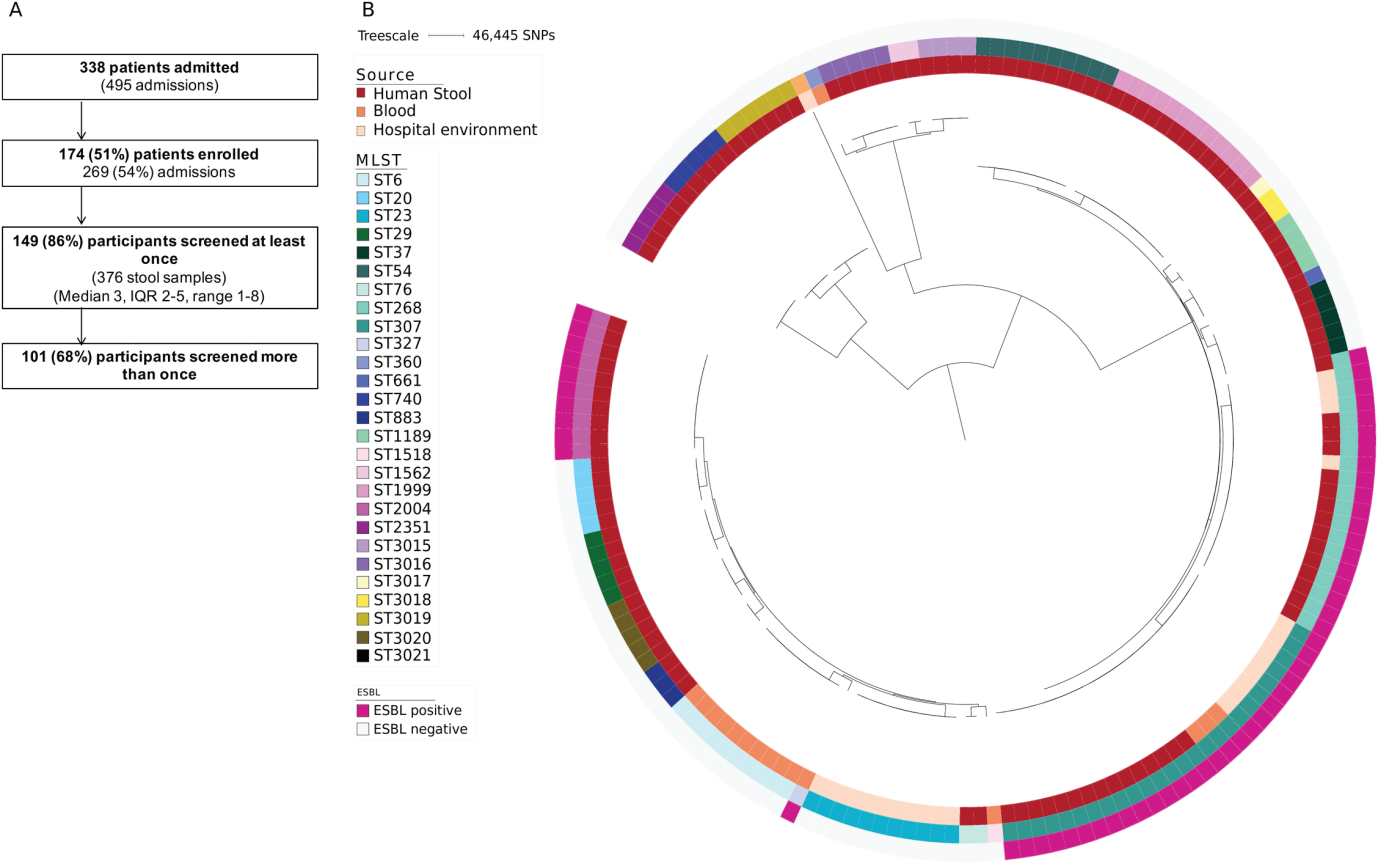
Patient recruitment and phylogeny of healthcare-associated *Klebsiella pneumoniae* isolates. *A*, Patients recruited and samples collected from study participants. *B*, Maximum likelihood tree of isolates from patient stool, blood, and the hospital environment based on single-nucleotides polymorphisms in the core genome. Abbreviations: ESBL, extended-spectrum beta-lactamase; IQR, interquartile range; MLST, multilocus sequence type; SNP, single-nucleotide polymorphism

Of 338 patients (51%) admitted to the 2 study wards between May 2015 and November 2015, 174 were recruited and agreed to provide stools for culture (Figure 1A). The 174 cases had a median stay of 16 days (interquartile range [IQR] 7–27 days) and were admitted a median of once (IQR 1–2, total 281 admissions). Stool samples were obtained from 149/174 patients (376 stools; median, 3 [IQR 2–5] per case), with 101 patients providing 2 or more samples. *K. pneumoniae* was isolated from 23 stools from 17/149 (11%) patients, 3 of whom (2%) carried ESBL-producing *K. pneumoniae*, a similar rate to that reported previously [24]. A total of 922 environmental swabs were taken from patient areas, medical equipment, and the wider environment over the 6-month study period. *K. pneumoniae* was isolated from 18 (2%) swabs from 7 locations (3 single rooms/bathrooms during an initial point prevalence survey and 3 single rooms/bathrooms plus a 2-bedded bay thereafter), each positive on 1 occasion. Six positive locations were in ward A and 1 was in ward B. This low rate of contamination is consistent with previous studies reporting 0%–5% positivity during routine surveillance [25, 26]. Bloodstream infection caused by *K. pneumoniae* occurred in 5 patients on the 2 study wards during an extended 2-year period (May 2014 to May 2016), including 1 case during the 6-month study. Seven *K. pneumoniae* cultures from the 5 patients had been stored and were available for sequencing (Supplementary Table 1).

### Genomic Analysis of Healthcare-associated Isolates

We sequenced 122 *K. pneumoniae* colonies (termed isolates) picked from primary culture plates from 23 stools/17 participants (median, 5 isolates per stool; range, 1–15). These were assigned to 21 STs (Figure 1B and Supplementary Table 1). Most patients (15/17) carried a single ST, with the 2 remaining cases each carrying 3 STs (Supplementary Table 1). No 2 patients carried the same ST, indicating an absence of patient-to-patient transmission. Pairwise analysis of SNPs in the core genome of isolates from the same patient/same ST demonstrated a median (range) difference of 1 (0–30) SNPs for the 17 patients.

We sequenced 24 isolates from 18 environmental swabs. These belonged to a more restricted population of 4 STs (Figure 1B and Supplementary Table 1). Integration of genetic and epidemiological data suggested 2 broad environmental contamination events. A cluster of 11 *K. pneumoniae* ST23 isolates were cultured from 2 adjacent single rooms and 1 more distant single room in ward A, all positive on a single date in April 2014. Pairwise core genome SNP analysis demonstrated a median (range) difference of 2 (range, 0–5) SNPs after removing 2 outliers (≥45 SNPs different). A second cluster of 8 *K. pneumoniae* ST307 isolates were cultured from 2 adjacent single rooms in ward A and 1 bedside in a 2-bedded bay in ward B, all positive on a single date in September 2015. The 8 isolates were identical at the core genome level. In addition, *K. pneumoniae* ST268 and ST3021 were each isolated once from different single rooms. The ST3021 isolate was cultured from the same room and day as an ST23 isolate, suggesting a wider contamination event and/or inadequate cleaning.

**Figure 2.**
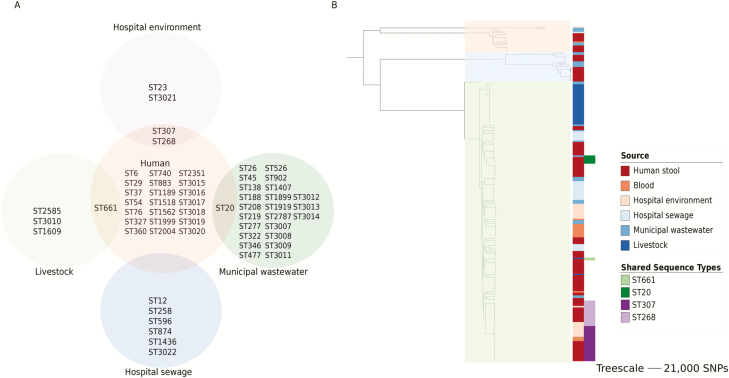
Relatedness of *Klebsiella pneumoniae* isolated from different sources. *A*, Distribution of multilocus sequence types (STs) by source of isolation. *B*, Maximum-likelihood core genome phylogeny of *K. pneumoniae* from humans, the hospital environment, hospital sewage, livestock, and municipal wastewater from the East of England. The 3 clades are highlighted in orange (KpI), blue (KpII), and green (KpIII). The 2 right-hand columns (from left to right) show isolate source and those cases where STs were the same for both human and nonhuman isolates. Abbreviation: SNP, single-nucleotide polymorphism.

**Figure 3.**
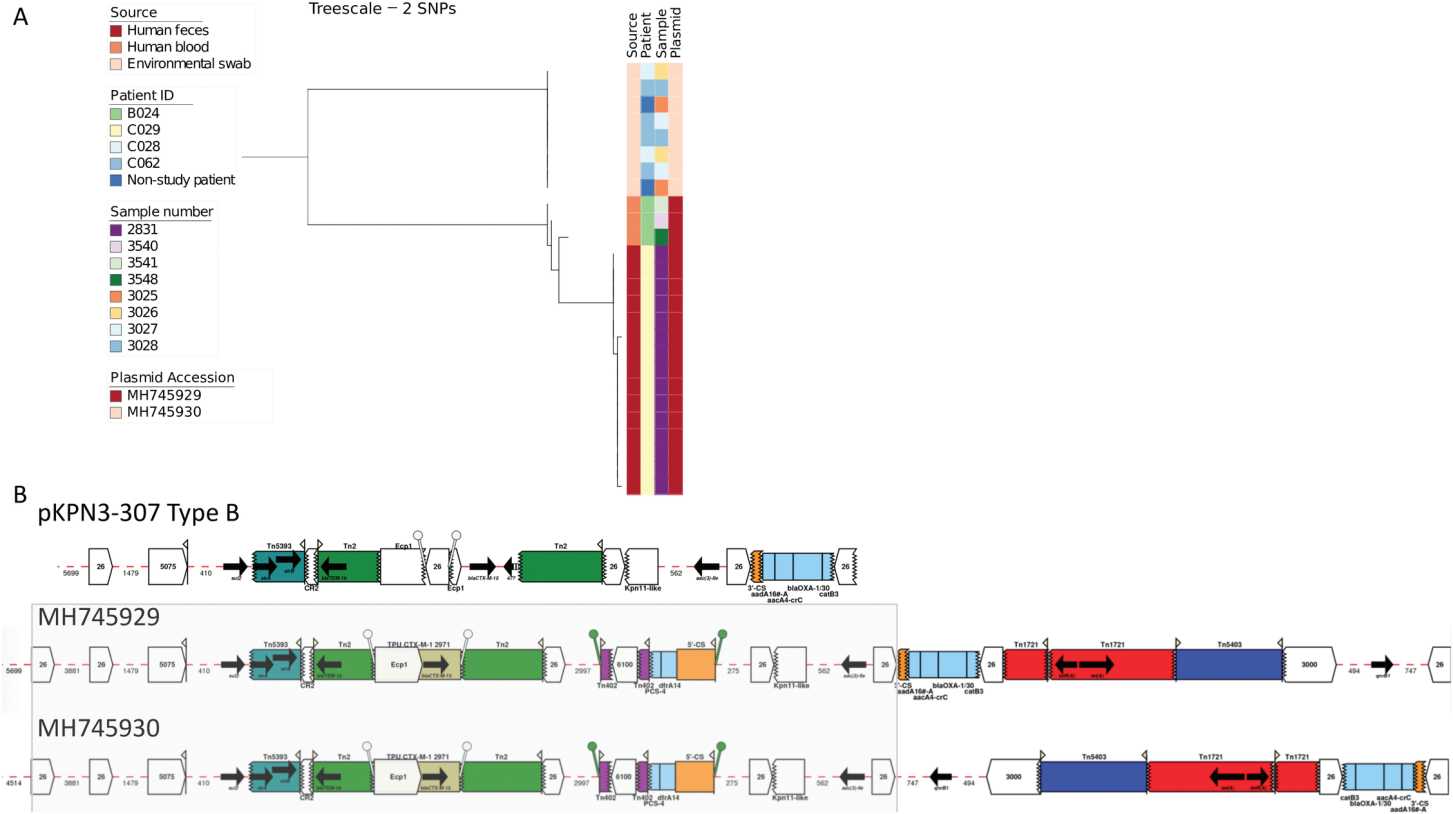
Phylogeny of sequence type (ST) ST307 *Klebsiella pneumoniae* isolates and characterization of plasmids present in isolates from patients and their environment. *A*, Maximum likelihood tree of ST307 *K. pneumoniae* isolates from patient stool, blood, and the hospital environment based on single-nucleotide polymorphisms in the core genome after removal of recombination. *B*, Antimicrobial resistance genes and associated mobile elements in the pKPN3-307 Type B (GenBank KY271405.1) reference plasmid and in the human (GenBank MH745929) and environmental plasmids (GenBank MH745930). The shared 31 533-bp antimicrobial resistance region containing the *bla*_*CTX-M-15*_ element is highlighted in gray. Arrows indicate the orientation of features, with the forward direction defined as the direction of transcription for genes, toward the main part of the attC site for cassettes, in integrons toward attI for 5’ flanking regions, away from the cassette array for 3’ flanking regions, relative to the direction of transcription of the transposase gene for insertion sequences and transposons (Tn) (ie, inverted repeat left to inverted repeat right) and to the direction of the reverse transcriptase for Group II introns. The missing end of a feature is shown by a zig-zag line. Abbreviation: SNP, single-nucleotide polymorphism.

Comparison of isolates from stool and the environment showed that 2 STs (ST268 and ST307) were identified from both sources (Figure 2A). ST268 was isolated from a single stool from a patient (D034) and their environment in August 2015 and October 2015, respectively. Patient D304 had 17 admissions to the hematology day unit and 2 transfers to ward A during the period between the positive stool and environment sample. The most closely related stool–environmental ST268 isolate pair had highly similar core genomes (1 SNP different), which is consistent with recent patient–environment transmission. ST307 was isolated from stool in August 2015 from a patient (C029) in a single room on ward B and 18 days later from the environment of 2 rooms in ward A and 1 room in ward B (a different room from the index case). The most closely related stool and environmental ST307 isolate pair was 64 core genome SNPs different (see Figure 3A for phylogeny), which is not consistent with recent patient–environment transmission.

We sequenced 16 *K. pneumoniae* isolates from 7 blood cultures/5 patients (1 isolate from each of the archived collection for 6 cultures/4 patients before or after the 6-month study and 10 primary plate colonies from the case that occurred during the study). These belonged to 5 STs, with 1 ST per patient. Four of the 5 STs were not identified in any other patient during the 6-month study, including the ST isolated from the patient who was bacteremic in this period. The exception was ST307, isolated from a patient (B024) with a bloodstream infection at 2 time points in September 2014 and December 2014 (at least 5 months before the prospective study began). Blood culture isolates from September and December differed by 4 SNPs, indicating relapse or reinfection with the same strain. Comparison between these and the prospective study isolates showed that the earlier blood culture isolates were related to the ST307 patient C029 stool isolates (13 SNPs; range, 12–15) and less closely to the ST307 environmental isolates (54 SNPs; range, 52–57; Figure 3A). This is indicative of either a persistent environmental reservoir of ST307 *K. pneumoniae* or ongoing transmission between unsampled patients. Sequencing and pairwise core genome analysis of 10 colonies from a single blood culture from 1 patient (B022, ST6) demonstrated a median (range) difference of 1 (0–2) SNP. This patient did not provide a stool sample.

### Isolation of *K. pneumoniae* From Livestock, Retail Meat, and Sewage

**Figure 4.**
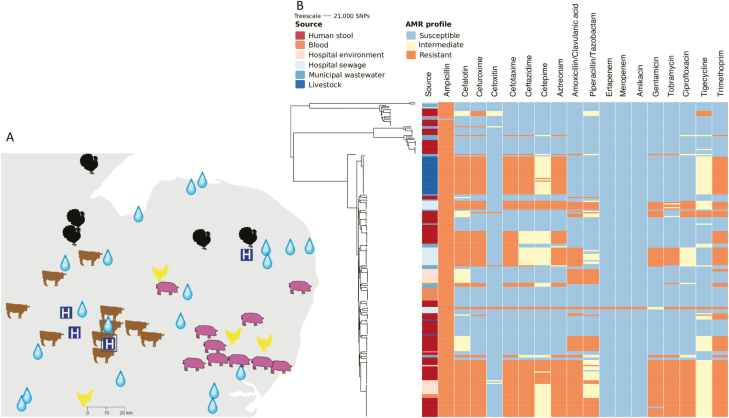
Sampling locations and phenotypic antimicrobial susceptibility across the *Klebsiella pneumoniae* phylogeny*. A*, Map of the East Anglia region of the United Kingdom, showing the locations of the farms (images indicate the animal species), wastewater treatment plants (water drops), and hospitals in the region (indicated by a white “H” in a blue square). The hospital where the clinical and environmental isolates were collected is shown with H surrounded by a square. Adapted from Gouliouris et al [29]. Copyright 2018 Gouliouris et al. Adapted with permission. *B*, Maximum likelihood core genome phylogeny of 249 *K. pneumoniae* from human stool, blood, livestock, municipal wastewater, and hospital sewage (left) and their phenotypic antimicrobial susceptibility (right). The first vertical column shows isolate source and the remainder, the susceptibility to 19 antimicrobial drugs. Abbreviation: SNP, single-nucleotide polymorphism.

The number of sampling points at each farm was maximized by taking pooled samples containing up to 50 aliquots of freshly passed fecal material from each major area in a given farm (eg, a pen). Culture of 136 pooled fecal samples from 29 livestock farms (Figure 4A) demonstrated that 6 (4%) samples from 4 (14%) farms (1 dairy cattle and 3 turkey) were positive for *K. pneumoniae* (Supplementary Table 1). Culture of 97 prepackaged fresh meat products from 11 countries purchased at 11 major Cambridge supermarkets were all negative for *K. pneumoniae* (Supplementary Table 2). Longitudinal sampling of sewage (4 samples over 16 months) at the study hospital (Figure 4A) resulted in *K. pneumoniae* being identified in 3/4 samples. A survey of 20 municipal wastewater treatment plants (Figure 4A) led to the recovery of *K. pneumoniae* from 17/40 water samples (11 untreated and 6 treated wastewater), taken from 12/20 treatment plants, with 5/12 plants releasing *K. pneumoniae* into the environment. Multiple colonies were selected from each positive sample for sequencing.

### Genome-based Comparison of *K. pneumoniae* From the Hematology Ward and Elsewhere

We sequenced 87 *K. pneumoniae* isolates from livestock (32 isolates), municipal wastewater treatment plants (28 isolates), and hospital sewage (27 isolates). From this we identified 4 STs from livestock isolates, 24 STs from municipal wastewater, and 6 STs from hospital sewage (Figure 2A). For the livestock isolates, 2 STs (ST1609 and ST661) were identified from a single cattle farm, and 1 ST (ST3010) was isolated from all 3 turkey farms together with 1 colony of its single locus variant (SLV) from 1 of these (ST2585). All isolates from 3 turkey farms (including the SLV) were a median (range) of 12 core genome SNPs (range, 0–17) different, suggesting linkage between farms. Of these, 2 farms were located approximately 24 miles apart and the third was approximately 82 miles and 66 miles from the 2 other farms, respectively. All 3 farms were owned by the same company, and it is possible that transmission may have occurred as a result, although the route of transmission is unknown. STs were compared between isolates from the patient cohort, their ward environment, livestock, and municipal and hospital waste (Figure 2A). There was no overlap in STs from the different sources with 2 exceptions (ST661 from patient stool and livestock, and ST20 from patient stool and municipal wastewater). The 2 ST611 isolates differed by 2641 core genome SNPs, and the 6 ST20 isolates differed by a median (range) of 849 (848–851) SNPs. This indicates that isolates from different sources that belonged to the same ST were not closely related.

A maximum-likelihood phylogenetic tree of the 249 study isolates (hematology ward [n = 162], livestock/sewage/wastewater [n = 87]) was constructed based on 499 378 core gene SNPs (Figure 2B). This demonstrated high genetic diversity and 3 distinct populations (208 KpI [*K. pneumoniae*], 22 KpII [*Klebsiella quasipneumoniae*], and 19 KpIII [*Klebsiella variicola*]) isolates) (Figure 2B), consistent with previous descriptions [1]. Each clade contained isolates from human stool and wastewater. Isolates from the hospital environment and blood were confined to KpI and KpIII, while isolates from cattle and turkeys resided in different clades (turkey in KpI, cattle in KpII).

### Antibiotic Resistance in *K. pneumoniae* From the Hematology Ward and Elsewhere

Phenotypic antibiotic susceptibility of the 249 isolates to 19 antibiotics is summarized in Figure 4B. Resistance to meropenem and ertapenem was detected for 2 isolates from hospital sewage, both of which contained *bla*_KPC-2_. No colistin resistance genes (MCR-1, MCR-1.2, MCR-2, MCR-3, MCR-4, MCR-5) were detected in any of the isolates. Colistin resistance in *K. pneumoniae* can also occur through modification of lipopolysaccharides that arise from mutations in the *pmrA*/*pmrB* and *phoP*/*phoQ* two-component regulatory systems. We identified the L96P mutation in *phoQ* associated previously with colistin resistance [27] in 3 isolates recovered from a single wastewater treatment plant (accession numbers ERR985164, ERR985165, ERR985166). This indicates the importance of wastewater treatment plants as a source of resistance variants. Almost half of all isolates had an ESBL phenotype (118/249, 47%). Nearly all ESBL *K. pneumoniae* (n = 113) carried *bla*_*CTX-M-15*_, the remainder carrying *bla*_*CTX-M-1*_ (n = 3) or *bla*_KPC-2_ and *bla*_SHV-12_ (n = 2). Isolates positive for *bla*_*CTX-M-15*_ were distributed across 12 STs and in all reservoirs tested, while *bla*_*CTX-M-1*_ and *bla*_KPC-2_ were reservoir specific and restricted to ST277 and ST258, respectively (Supplementary Table 1).

Plasmid analysis for the 113 *bla*_*CTX-M-15*_ positive isolates was initially performed based on comparison between contigs containing the gene and the National Center for Biotechnology Information (NCBI) nucleotide database. This demonstrated close similarity to several reference plasmids, against which short-read data for the 113 isolates were then mapped. All isolates from turkey farms contained a *bla*_*CTX-M-15*_ plasmid with high sequence similarity (ID >99%, coverage >99%) to pKpN01-CTX [28]. Wastewater isolates that belonged to ST902 (municipal plant) and ST1436 (hospital sewage) showed high sequence match (ID >99%, coverage >98%) to pKPN3-307 type A plasmid [30]. All ST268 isolates from stool and the ward environment mapped (ID >99%, coverage >99%) to the plasmid pCTXM15 (GenBank accession CP016925.1), consistent with these being associated with patient–environmental transmission. By contrast, while ST307 isolates from blood and stool mapped to IncFIB(K) pKPN3-307 type B (GenBank KY271405.1; ID >97%, coverage >99%), ST307 isolates from the hospital environment were not positive for this plasmid and no plasmid reference was identified in the NCBI nucleotide database. This is consistent with these not being associated with patient–environment transmission. Long-read sequencing of 1 bloodstream and 1 environmental *bla*_CTX-M-15_ ST307 isolate showed that both contained an IncFIB(K) plasmid (GenBank MH745929 and MH745930, respectively). The environmental plasmid had only 78% coverage (ID >99%) against the human plasmid; however, this shared sequence included a 31 533-bp antimicrobial resistance region containing the *bla*_*CTX-M-15*_ element (Figure 3B and Supplementary Table 3). Mapping of all ST307 *bla*_CTX-M-15_ isolates to these 2 plasmids showed that clinical isolates contained MH745929 and the environmental isolates contained MH745930 (Figure 3A).

All *bla*_*CTX-M-15*_ plasmids encoded a 13.4-kb region containing multiple antimicrobial resistance genes including *bla*_*CTX*-M-15_, *bla*_*TEM*_, *strA*, *strB*, and *sul2* (see Figure 3B and AMR_Cluster1 in Supplementary Table 4). In total, 88/113 *bla*_*CTX-M-15*_ positive isolates from humans, the hospital environment, livestock, and wastewater had high coverage mapping (>99%) and sequence ID (>99%) to this cluster, which shared synteny with an antimicrobial resistance region found previously in other *K. pneumoniae* plasmids [30]. In addition, 17 isolates from the hospital sewer and humans shared a 7.2-kb resistance cluster (see AMR_Cluster2 in Supplementary Table 3), containing *bla*_*CTX-M-15*_ and *aph(3)* (encoding aminoglycoside resistance).

Analysis of *bla*_*CTX-M-1*_ and *bla*_KPC-2_ positive isolates demonstrated that the 3 *bla*_*CTX-M-1*_ isolates (ST277, from wastewater) mapped with high similarity to the p369 plasmid (GenBank accession KT779550; ID >99%, coverage >99%) [31], and the 2 carbapenemase-producing *K. pneumoniae* (*bla*_KPC-2_) isolates (ST258) from hospital sewage showed >99% mapping coverage and >99% sequence ID to the whole sequence of KPC reference plasmid pKpQIL-UK [32].

### Virulence Genes in *K. pneumoniae* From Different Reservoirs

The diversity of loci encoding capsule serotypes and the presence of specific virulence genes were identified in the 249 study isolates. Supplementary Table 1 provides a detailed description of capsule typing results. In brief, 43 capsule loci were identified (Supplementary Figure 1A), with 4 capsule loci with the same *wzi* gene allele detected in more than 1 reservoir (Supplementary Figure 1B). The *rmpA* gene (encoding a hypermucoid phenotype associated with increased capsule production) was identified in 1 isolate (Supplementary Table 1). Colibactin and aerobactin were only found in environmental isolates, and salmochelin was not detected in any study isolate. Yersiniabactin and ICE*Kp* elements were identified in hospital sewage, wastewater, humans, and the hospital environment, but ICE*Kp* types and yersiniabactin loci were predominantly reservoir- and MLST-specific (Supplementary Table 1). ICE*Kp*10 was the only element that carried genes encoding yersiniabactin (*ybt*1), colibactin, and aerobactin and was present in ST23 isolates from the ward environment.

## DISCUSSION

Here, we identified transmission of *K. pneumoniae* between patients and their ward environment and several broad environmental contamination events. A previous study suggested that *K. pneumoniae* is more transmissible than *Escherichia coli* [33], but we did not capture episodes of patient-to-patient transmission. The extensive genetic diversity of *K. pneumoniae* isolated from patient stool is consistent with previous studies [14, 24]. Within-host diversity was limited, with 2/17 cases carrying more than 1 lineage and little diversity within the same lineage in a given host. We isolated *K. pneumoniae* from turkey, dairy cattle, and wastewater, as described previously [34–38], but found no evidence to indicate that livestock or wastewater acted as a recent reservoir for *K. pneumoniae* isolated from patients. By contrast, we identified transmission of *K. pneumoniae* between farms.

Highly related plasmids carrying *bla*_*CTX-M-15*_ were identified in isolates from the healthcare setting and in isolates from nonhealthcare settings, but we found no evidence for sharing of the same plasmid in healthcare and nonhealthcare isolates. This suggests that livestock, wastewater, and hospital sewage in this region were not a direct source of *bla*_*CTX-M-15*_ plasmids in humans. Wastewater treatment plants were ineffective in eliminating *K. pneumoniae* from wastewater, leading to downstream environmental contamination, which may contribute to the spread of antimicrobial resistance. Livestock isolates lacked virulence genes for yersiniabactin, salmochelin, aerobactin, and colibactin, suggesting that livestock do not play a role in disseminating these virulence factors. Yersiniabactin was found in isolates from patients, municipal wastewater, and hospital sewage, but the presence of an identical genetic locus (*ybt* 9) was only identified in ST268 isolates from a patient and their environment.

Our study has several limitations. We only recruited half of patients admitted to the 2 hematology wards, and alternative sources such as sinks and taps and patient food and water were not investigated. Overall, 17/149 (11%) patients were positive for stool carriage of *K. pneumoniae,* which is similar to that reported in Australia [24] but lower than a prevalence rate reported from the United States [15]. This low rate of recovery reduced the power to detect genetic relatedness between healthcare- and nonhealthcare-associated isolates. Although we found no evidence for zoonotic transmission, this may not reflect the situation elsewhere, particularly where people and livestock live in closer proximity.

In conclusion, our findings support the continued focus on reducing environmental *K. pneumoniae* reservoirs in hospital settings. Within the limits of detection of the study, our findings do not support the suggestion that *K. pneumoniae* or their mobile genetic elements encoding antibiotic resistance are commonly acquired from livestock.

## Supplementary Data

Supplementary materials are available at *Clinical Infectious Diseases* online. Consisting of data provided by the authors to benefit the reader, the posted materials are not copyedited and are the sole responsibility of the authors, so questions or comments should be addressed to the corresponding author.

ciz174_suppl_Supplementary_Table_1

ciz174_suppl_Supplementary_Table_2

ciz174_suppl_Supplementary_Table_3

ciz174_suppl_Supplementary_Table_4

ciz174_suppl_Supplementary_data

## Notes

***Author contributions.***Study design: C. L., T. G., and S. J. P. Development of study protocols: C. L. and T. G. Collection of wastewater and farm samples: C. L. and T. G. Sourcing and microbiological culture of retail meat samples: N. H., T. G., and M. H. Obtaining access to farms and abattoirs: J. H. G., P. W., M. R., and M. H. Bacterial identification, susceptibility testing, and phenotypic testing: C. L., T. G., B. B., and P.N. Bioinformatics analyses and interpretation: C. L., D. M., and D. J. Figure production: C. L. Writing the manuscript: C. L. and S. J. P. Responsibility for supervision and management of the study: J. P. and S. J. P. All authors read and approved the final manuscript.

***Acknowledgments.***The authors thank the Wellcome Sanger Institute core library construction, sequence, and informatics teams and the Pathogen Informatics team. The authors thank the staff at farms, abattoirs, and wastewater treatment plants for assistance in sample collection, and Elizabeth Lay for laboratory support during the meat survey. The authors thank Lois Chaparadza and Rosie Swayne for support during the ward study. The authors also thank Alexander M. Wailan for advice on plasmid biology. The flocked swabs used in the meat survey were donated by Copan Italia spa. The study protocol was approved by the Cambridge University Hospitals NHS Foundation Trust Research and Development Department (ref. A093285); the Department of Veterinary Medicine, University of Cambridge (study CR112); and the National Research Ethics Service East of England Ethics Committee (ref. 12/EE/0439 and 14/EE/1123). Sequence data for all isolates have been submitted to the European Nucleotide Archive (www.ebi.ac.uk/ena) under study accession numbers PRJEB18814 and PRJEB19918 (all human, environmental, and hospital sewage *Klebsiella pneumoniae*), PRJEB8771 and PRJEB8776 (*K. pneumoniae* from livestock), and PRJEB8766 and PRJEB8770 (*K. pneumoniae* from wastewater) with the accession numbers for individual isolates listed in Supplementary Table 1.

***Disclaimer.***The views expressed in this publication are those of the author(s) and not necessarily those of the Department of Health or Wellcome Trust.

***Financial support.***This work was supported by grants from the Health Innovation Challenge Fund (grant numbers WT098600 and HICF-T5-342),a parallel funding partnership between the UK Department of Health and the Wellcome Trust. This project was also supported by a grant awarded from the Wellcome Trust (098051). T. G. is a Wellcome Trust Research Training Fellow (103387/Z/13/Z). C. L. is a Wellcome Trust Sir Henry Postdoctoral Fellow (110243/Z/15/Z). D. J. is funded by the Wellcome Trust (grant 098051).

***Potential conflicts of interest.***S.J.P. and J.P. report personal fees from Next Gen Diagnostics outside the submitted work. All other authors report no potential conflicts. All authors have submitted the ICMJE Form for Disclosure of Potential Conflicts of Interest. Conflicts that the editors consider relevant to the content of the manuscript have been disclosed.
