# Supplementary material for: A One Health Study of the Genetic Relatedness of Klebsiella pneumoniae and Their Mobile Elements in the East of England
Source: Clin Infect Dis. 2019 Mar 7;70(2):219–26. doi: 10.1093/cid/ciz174 (PMC6938978; doi:10.1093/cid/ciz174)
Supplement: ciz174_suppl_Supplementary_data [file ciz174_suppl_supplementary_data.docx]

**SUPPLEMENTARY MATERIALS**

**A One Health study of the genetic relatedness of *Klebsiella pneumoniae* and their mobile elements in the East of England**

Catherine Ludden, Danesh Moradigaravand, Dorota Jamrozy, Theodore Gouliouris, Beth Blane, Plamena Naydenova, Juan Hernandez-Garcia, Paul Wood, Nazreen Hadjirin, Milorad Radakovic, Charles Crawley, Nicholas M. Brown, Mark Holmes, Julian Parkhill, Sharon J. Peacock

**SUPPLEMENTARY METHODS**

*Longitudinal survey of K. pneumoniae carriage and bloodstream infection*

A prospective longitudinal study was conducted in two adult hematology wards at the Cambridge University Hospital NHS Foundation Trust (CUH) between May and November 2015. Patients were enrolled following informed written consent, after which stool samples were requested on the day of admission, every week thereafter and at discharge and cultured for *K. pneumoniae*. Blood cultures were taken by treating clinicians based on clinical need and processed by the routine diagnostic microbiology laboratory. All patients with positive *K. pneumoniae* blood cultures on the two hematology wards between May 2014 to May 2016 were identified and isolates from each case were retrieved from the microbiology laboratory freezer archive.

*Sampling the ward environment*

A point prevalence survey was performed 2 weeks before the start of patient recruitment to establish baseline levels of environmental contamination and to test the isolation methodology for ESBL-negative *K. pneumoniae* (from non-selective agar) and ESBL-positive *K. pneumoniae* (see bacterial culture below for details). Environmental sites (approximately 10 cm × 10 cm) were sampled using flocked swabs (FLOQSwabs, Copan Italia spa, Brescia, Italy), which contain a sponge with a detergent neutralising solution and were immediately placed in 3 ml of Brain Heart Infusion (BHI broth). Individual swabs were taken from each of four high-frequency touch bedside areas and 3 high-frequency touch areas in the bathroom/toilet of every in-patient room. Swabs were also taken from: i) communal bathrooms/toilets, ii) medical equipment (computers-on-wheels and nursing hand-held devices), and iii) non-touch areas (air vents and HEPA filter vents). During the six-month longitudinal study, sampling was performed every two weeks of the communal bathrooms, toilets, non-touch surfaces (air vents and HEPA filters) and a range of medical devices in the two in-patent wards. In addition, two pooled swabs were taken from the bedside (bedside locker, table and bedrails) and bathroom areas (toilet and shower) on the day of discharge for patients who did not provide a stool sample. The hematology day unit was swabbed throughout at the start and midpoint of the study.

*Wastewater sampling*

A cross-sectional survey was conducted between June 2014 and January 2015 to isolate *K. pneumoniae* from raw and treated wastewater collected from 20 municipal wastewater treatment plants in the East of England. Of these, 10 were located downstream of acute hospitals and 10 did not directly receive hospital waste. Wastewater was also sampled from the main sewer of CUH on four spaced occasions between September 2014 and December 2015. At each sampling point, two consecutive grab samples of 0.5 L each were collected and mixed into 1 L sterile bottles containing 18 mg sodium thiosulphate (Sigma-Aldrich, Poole, UK), with the exception of CUH where a single 1 L wastewater sample was obtained on each occasion.

*Sampling from livestock farms and meat*

A cross-sectional survey was conducted between August 2014 and April 2015 to isolate *K. pneumoniae* at 20 livestock farms (10 cattle & 10 pig) in the East of England. A pooled sample of around 50g of freshly passed fecal material was collected from every major area of each farm using a sterile scoop (Sterilin™ X400, Thermo Fisher Scientific, Loughborough, UK). A median of 4 samples (range 1-5) were taken from each cattle farm, and a median of 4.5 samples (range 3-9) taken from each pig farm, resulting in a total of 85 pooled samples (34 cattle and 51 pig). In addition, cecal contents were collected from 2 deceased pigs at the time of necropsy. Poultry reared at nine farms (4 chicken and 5 turkey) in the East of England were sampled at two abattoirs between February and April 2015. Two sample types were taken for each farm: (i) pooled faeces with a total weight of around 50 g from 10 - 20 transportation crates immediately after the animals were removed; (ii) pools of cecal material from up to 10 birds after slaughter. A median of 4 (range 2-4) cecal pools and a median of 4 (range 3-4) fecal pools were collected from animals from each chicken farm. A median of 1.5 (range 1-2) cecal pools and a median of 2.5 (range 2-3) fecal pools were collected from animals from each turkey farm. This resulted in a total of 49 pooled samples (29 chicken and 20 turkey).

In April 2015, 97 retail meat samples (beef 15, chicken 30, pork 42, turkey 7, venison 1, mixed minced pork and beef 1) were purchased from 11 supermarkets in Cambridge, UK, with 5-16 samples collected from each supermarket (Supplemental Table S2). Country of origin was recorded and where multiple countries/regions were stated on the packaging, all names were recorded.

*Bacterial culture*

Stools were directly plated onto Brilliance UTI Chromagar (Oxoid, Basingstoke, UK) and Brilliance™ ESBL agar (Oxoid, Basingstoke, UK). Enrichment cultures were also performed to detect ESBL-producing *K. pneumoniae* by adding approximately 0.2 g of stool to 10 mL of Tryptic Soy Broth (Sigma, Dorset, UK) containing cefpodoxime 1 μg/mL (Oxoid), vortexed, and incubated with shaking at 150 rpm at 37 °C in air overnight. The following day, 200 μL of suspension was inoculated onto Brilliance™ ESBL agar (Oxoid, Basingstoke, UK). Environmental swabs were processed within 4 hours. These were placed in BHI broths, vortexed for 30 seconds and then incubated at 37°C shaking at 150 rpm for 18-24 hours, after which 100 μl of BHI broth was inoculated onto Chromocult Agar (VWR) and Brilliance™ ESBL agar during the point prevalence study and the latter agar alone thereafter. Blood culture sets consisting of three bottles (aerobic, anaerobic and FAN, BacT/ALERT, bioMérieux) were obtained peripherally and/or centrally as clinically indicated and cultured by the diagnostic laboratory. For wastewater, 1 mL of triplicate serial ten-fold dilutions, 10 mL of treated and untreated wastewater and 100 mL of treated wastewater were concentrated using the filtration technique onto 0.45 μm pore size filter membranes (S-Pak, Merck Millipore, Darmstadt, Germany). Membranes were placed onto the surface of Chromocult Agar (VWR) and Brilliance™ ESBL agar (Oxoid, Basingstoke, UK). Pooled fecal samples from livestock farms were diluted 1:1 with sterile phosphate-buffered saline, mixed vigorously and 100 μl plated onto Chromocult Agar and Brilliance™ ESBL agar. Enrichment cultures were also performed for ESBL-producing *K. pneumoniae* by adding 1 ml of fecal preparation to 9 ml of tryptic soy broth containing 20 µg cefpodoxime and incubated at 37°C shaking at 150 rpm for 24 hours before plating 100 μl onto Brilliance™ ESBL agar.

Preparation and culture of meat samples followed the European standard ISO 6887–2:2003. A 5 g sample of meat was aseptically removed from packaging, added to 45 ml peptone broth and homogenised using a Stomacher® paddle blender (Stomacher®80 Laboratory System, Seward Ltd, UK) for two minutes. Samples were transferred into 50 ml Falcon™ tubes and incubated in a shaking incubator for 24 hours at 150 rpm at 37°C. After incubation, all samples, were plated onto *Brilliance*™ ESBL agar. In addition, swabs were obtained from whole chicken carcasses and incubated in 3 ml brain heart infusion (BHI) broth (FlOQSwabs™, Copan Italia spa, Brescia, Italy) in a shaking incubator for 24 hours at 150 rpm at 37°C. Following incubation, 100μL was plated onto *Brilliance*™ ESBL agar. All Chromocult and Brilliance™ ESBL agar plates were incubated at 37**°**C in air for 24 hours and 48 hours, respectively.

*Bacterial identification and antimicrobial susceptibility testing*

Multiple bacterial colonies suspected to be *K. pneumoniae* based on colonial morphology and color were selected from all stool, environmental, wastewater, livestock and meat samples. For blood cultures positive for *K. pneumoniae* during the six-month study, 10 colonies were picked from positive primary plates for each positive blood culture set. For blood cultures positive for *K. pneumoniae* in the 12 months before (May 2014 –May 2015) and six-months after the study (November 2015-May 2016), one colony was obtained from culture of the freezer archive. One *K. pneumoniae* colony was selected from every positive environmental sample taken during the pilot study and five from each positive pooled environmental sample taken during the six-month clinical study. Up to 10 colonies were picked from samples taken on each farm and wastewater treatment plant. *K. pneumoniae* was confirmed by mass spectrometry (MALDI-TOF MS, Bruker Daltonics, Coventry, UK). Antimicrobial susceptibility testing was determined using the N206 card on the Vitek 2 instrument (bioMérieux, Marcy l’Étoile, France) calibrated against EUCAST breakpoints.

*Isolates for sequencing, DNA extraction, sequencing and additional genomes*

The following *K. pneumoniae* colonies (termed isolates) were randomly selected for sequencing: up to 15 from every positive stool (5 ESBL negative and 10 ESBL positive, or 15 ESBL if no ESBL negative colonies were cultured); 10 from blood cultures positive for *K. pneumoniae* during the six-month study period and a single colony from freezer archives made from purity plates; one colony from every positive environmental sample taken during the pilot study, two colonies from every positive pooled environmental sample taken during the study period; and (due to scarcity) all presumptive *K. pneumoniae* (ESBL-negative and ESBL-positive) observed on cultures from livestock and wastewater. Bacterial genomic DNA was extracted using the QIAxtractor (QIAgen), according to the manufacturer's instructions. Library preparation was conducted according to the Illumina protocol and sequenced on an Illumina HiSeq2000 (Illumina, San Diego, CA, USA) with 125-cycle paired-end runs.

*Genome assembly, annotation and pan-genome analysis*

Taxonomic identity was assigned to all short reads and assemblies using Kraken [1]. Unless previously assembled, *de novo* assembly of short read data was performed using Velvet [2] and the quality of the sequences were checked by determining the length of each genome, number of contigs in the assembly and depth of coverage against a reference (*Klebsiella pneumoniae* Ecl8). Ten genomes were removed from further analysis based on quality metrics. *de novo* assembly of short read data for the remaining 249 isolates was performed as previously described [2, 3] and assemblies were annotated using Prokka [4], the output of which was used as input for the pan-genome pipeline Roary [5]. A core genome alignment was produced by Roary.

*MLST analysis and identification of antimicrobial resistance determinants, virulence factors and plasmids*

Multilocus sequence types (STs) were identified using Kleborate [6]. Novel alleles and MLST profiles were submitted to BIGSdb, Institut Pasteur for curation (<http://bigsdb.pasteur.fr/klebsiella/klebsiella.html)>. Isolates were screened for the presence of *Kp* integrative conjugative elements (ICEKp), yersiniabactin, colibactin, aerobactin and salmochelin using Kleborate [6]. Capsule locus and *wzi* locus typing was conducted with Kaptive [7]. The srst2 package [8] was used with a coverage cut-off of 90% to screen short read data for known antibiotic resistance genes using the ResFinder database. To study the mutations underlying colistin resistance, we extracted gene variants associated with resistance, including the two-component system (TCS) genes *phoPQ* and *pmrAB*, the repressor *mgrB* and the sensory component of the TCS *crrAB*, as reported previously [9-12]. Using the Comprehensive Antibiotic Resistance Database (CARD), we also screened all isolates for the presence of a group of mobile colistin resistance genes that encode the MCR family of phosphoethanolamine transferases (CARD ID:3004268) and the MCR gene variants MCR-1 (CARD ID:3003689), MCR-1.2 (CARD ID:3004194), MCR-2 (CARD ID:3004110), MCR-3(CARD ID:3004139), MCR-4 (CARD ID:3004325) and MCR-5 (CARD ID:3004332). All accessory genome regions were identified as described previously in [13], and contigs carrying resistance genes of interest were analyzed further to identify mobile genetic elements (MGEs) associated with resistance genes. All unique sequences were aligned against the NCBI nucleotide database using BLAST and close similarity to several reference plasmids was identified. To determine distribution of these plasmids amongst the analyzed isolates, mapping of short reads against the plasmid sequences was performed using srst2 with the default minimum coverage cut off of 90% [8]. *in silico* PCR was used to perform plasmid incompatibility group/replicon typing [14]. Two ST307 *bla*_CTX-M-15_ positive isolates (one from the environment (ERR1878502) and one from a patient (ERR1878490)) were selected for long-read sequencing. DNA was extracted for the two representatives using the Qiagen MagAttract HMW DNA kit (Qiagen, Valencia, CA, USA) and sequenced using the PacBio Sequel instrument (Pacific Biosciences, Menlo Park, CA, USA ; <http://www.pacificbiosciences.com>). De-multiplexed sequences reads were assembled using HGAP v4 with SMRT® Link v5.1.0 (<https://www.pacb.com/documentation/analysis-procedure-multiplexed-microbial-assembly-with-smrt-link-v510/>), circularized using Circlator v1.5.3 [19].and each assembly annotated using Prokka [4]. Fully assembled plasmids were compared using WebACT (<http://www.webact.org)> and BLASTn (<https://blast.ncbi.nlm.nih.gov>). Annotation of genes encoding antibiotic resistance and associated mobile elements was performed using the Galileo AMR online tool [16, 17].

**SUPPLEMENTARY REFERENCES**

1. Wood DE, Salzberg SL. Kraken: ultrafast metagenomic sequence classification using exact alignments. Genome Biology **2014**; 15(3): R46.

2. Zerbino DR, Birney E. Velvet: algorithms for de novo short read assembly using de Bruijn graphs. Genome Res **2008**; 18.

3. Page AJ, De Silva N, Hunt M, et al. Robust high-throughput prokaryote de novo assembly and improvement pipeline for Illumina data. Microbial Genomics **2016**; 2(8).

4. Seemann T. Prokka: rapid prokaryotic genome annotation. Bioinformatics **2014**; 30(14): 2068-9.

5. Page AJ, Cummins CA, Hunt M, et al. Roary: rapid large-scale prokaryote pan genome analysis. Bioinformatics **2015**.

6. Lam MMC, Wick RR, Wyres KL, et al. Frequent emergence of pathogenic lineages of *Klebsiella pneumoniae* via mobilisation of yersiniabactin and colibactin. bioRxiv **2017**.

7. Wyres KL, Wick RR, Gorrie C, et al. Identification of *Klebsiella* capsule synthesis loci from whole genome data. Microbial Genomics **2016**; 2(12).

8. Inouye M, Dashnow H, Raven LA, et al. SRST2: Rapid genomic surveillance for public health and hospital microbiology labs. Genome Med **2014**; 6(11): 90.

9. Olaitan AO, Morand S, Rolain J-M. Mechanisms of polymyxin resistance: acquired and intrinsic resistance in bacteria. Frontiers in Microbiology **2014**; 5: 643.

10. Cain AK, Boinett CJ, Barquist L, et al. Morphological, genomic and transcriptomic responses of Klebsiella pneumoniae to the last-line antibiotic colistin. Scientific Reports **2018**; 8: 9868.

11. Wright MS, Suzuki Y, Jones MB, et al. Genomic and Transcriptomic Analyses of Colistin-Resistant Clinical Isolates of Klebsiella pneumoniae Reveal Multiple Pathways of Resistance. Antimicrobial Agents and Chemotherapy **2015**; 59(1): 536-43.

12. Olaitan AO, Diene SM, Kempf M, et al. Worldwide emergence of colistin resistance in Klebsiella pneumoniae from healthy humans and patients in Lao PDR, Thailand, Israel, Nigeria and France owing to inactivation of the PhoP/PhoQ regulator mgrB: an epidemiological and molecular study. International Journal of Antimicrobial Agents **2014**; 44(6): 500-7.

13. Harris SR, Feil EJ, Holden MT, et al. Evolution of MRSA during hospital transmission and intercontinental spread. Science **2010**; 327.

14. Carattoli A, Bertini A, Villa L, Falbo V, Hopkins KL, Threlfall EJ. Identification of plasmids by PCR-based replicon typing. J Microbiol Methods **2005**; 63.

15. Wick RR, Judd LM, Gorrie CL, Holt KE. Unicycler: Resolving bacterial genome assemblies from short and long sequencing reads. PLOS Computational Biology **2017**; 13(6): e1005595.

16. Partridge SR, Tsafnat G, Coiera E, Iredell JR. Gene cassettes and cassette arrays in mobile resistance integrons. FEMS Microbiology Reviews **2009**; 33(4): 757-84.

17. Tsafnat G, Coiera E, Partridge SR, Schaeffer J, Iredell JR. Context-driven discovery of gene cassettes in mobile integrons using a computational grammar. BMC Bioinformatics **2009**; 10(1): 281.

**Supplementary Figure**


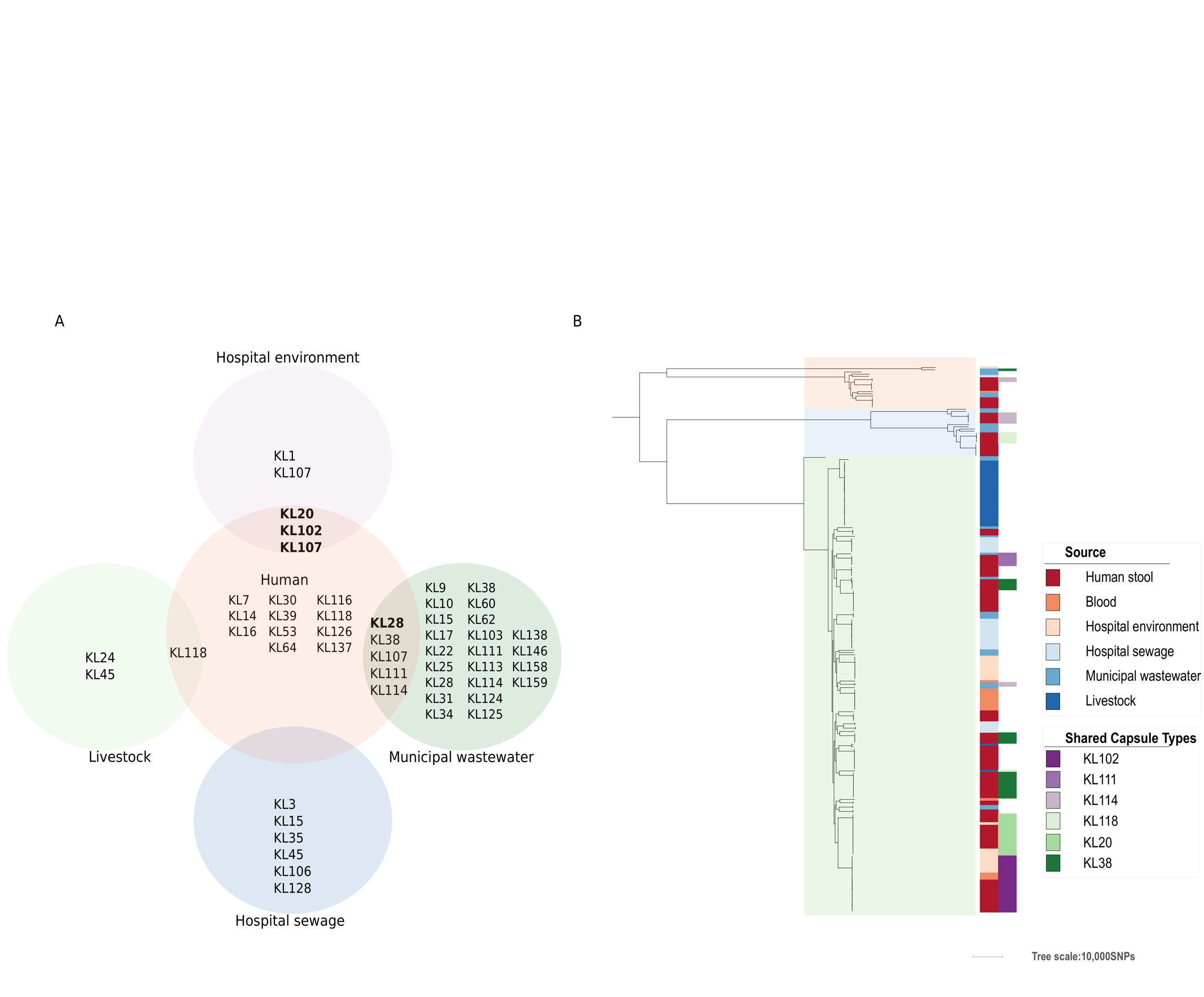


**Fig. S1. Capsule locus types by source of isolation and their distribution across the phylogeny
(A)** Distribution of capsule locus types (KL) by source. Capsule locus types highlighted in bold demonstrate capsule loci with the same *wzi* gene allele identified in both human and non-human isolates. **(B)** Maximum-likelihood core genome phylogeny of *K. pneumoniae* isolated from human stool, blood, the hospital environment, hospital sewage, livestock and municipal wastewater from the East of England. The columns (from left to right) show the source and capsule locus types present in both human and non-human isolates, referred to as shared capsule types. The three clades are highlighted in orange (KpI), blue (KpII) and green (KpIII).

**Supplementary Tables**

**Supplementary Table 1:** Details of all *K. pneumoniae* samples sequenced from human stool, blood, the hospital environment, hospital sewage, livestock and municipal wastewater from the East of England (see excel S1)

**Supplementary Table 2:** Details of meat samples collected as part of survey (see excel S2)

**Supplementary Table 3:** Sequence annotation of antimicrobial resistance regions identified on the human (MH745929) and environmental (MH745930) plasmids using the Galileo AMR online tool. **^*^**Arrows indicate the orientation of features. **^#^**In text annotations indicates that an incomplete copy of the feature is present. The dashed part of arrow indicates which end is missing. Annotations of partial features may not distinguish correctly between particular variants (see excel S3).

**Supplementary Table 4:** Details of common mobile elements denoted as “AMR_Cluster1” and “AMR_Cluster2” found in humans, the hospital environment, livestock, wastewater and hospital sewage (see excel S4).
